# Sulfated carboxymethyl cellulose and carboxymethyl κ-carrageenan immobilization on 3D-printed poly-ε-caprolactone scaffolds differentially promote pre-osteoblast proliferation and osteogenic activity

**DOI:** 10.3389/fbioe.2022.957263

**Published:** 2022-09-23

**Authors:** Sonia Abbasi-Ravasjani, Hadi Seddiqi, Ali Moghaddaszadeh, Mohammad-Ehsan Ghiasvand, Jianfeng Jin, Erfan Oliaei, Rommel Gaud Bacabac, Jenneke Klein-Nulend

**Affiliations:** ^1^ Department of Oral Cell Biology, Academic Centre for Dentistry Amsterdam (ACTA), Amsterdam Movement Sciences, University of Amsterdam and Vrije Universiteit Amsterdam, Amsterdam, Netherlands; ^2^ Department of Biomedical Engineering, Science and Research Branch, Islamic Azad University, Tehran, Iran; ^3^ Department of Mechanical Engineering, Amir Kabir University of Technology, Tehran, Iran; ^4^ Fiber and Polymer Technology, KTH Royal Institute of Technology, Stockholm, Sweden; ^5^ Medical Biophysics Group, Department of Physics, University of San Carlos, Cebu City, Philippines

**Keywords:** bio-functionalization, bone tissue engineering, carboxymethylated κ-carrageenan, finite element modeling, PCL (poly-є-caprolactone), pre-osteoblast, 3D-printed scaffold, sulfated carboxymethyl cellulose

## Abstract

The lack of bioactivity in three-dimensional (3D)-printing of poly-є-caprolactone (PCL) scaffolds limits cell-material interactions in bone tissue engineering. This constraint can be overcome by surface-functionalization using glycosaminoglycan-like anionic polysaccharides, e.g., carboxymethyl cellulose (CMC), a plant-based carboxymethylated, unsulfated polysaccharide, and κ-carrageenan, a seaweed-derived sulfated, non-carboxymethylated polysaccharide. The sulfation of CMC and carboxymethylation of κ-carrageenan critically improve their bioactivity. However, whether sulfated carboxymethyl cellulose (SCMC) and carboxymethyl κ-carrageenan (CM-κ-Car) affect the osteogenic differentiation potential of pre-osteoblasts on 3D-scaffolds is still unknown. Here, we aimed to assess the effects of surface-functionalization by SCMC or CM-κ-Car on the physicochemical and mechanical properties of 3D-printed PCL scaffolds, as well as the osteogenic response of pre-osteoblasts. MC3T3-E1 pre-osteoblasts were seeded on 3D-printed PCL scaffolds that were functionalized by CM-κ-Car (PCL/CM-κ-Car) or SCMC (PCL/SCMC), cultured up to 28 days. The scaffolds’ physicochemical and mechanical properties and pre-osteoblast function were assessed experimentally and by finite element (FE) modeling. We found that the surface-functionalization by SCMC and CM-κ-Car did not change the scaffold geometry and structure but decreased the elastic modulus. Furthermore, the scaffold surface roughness and hardness increased and the scaffold became more hydrophilic. The FE modeling results implied resilience up to 2% compression strain, which was below the yield stress for all scaffolds. Surface-functionalization by SCMC decreased *Runx2* and *Dmp1* expression, while surface-functionalization by CM-κ-Car increased *Cox2* expression at day 1. Surface-functionalization by SCMC most strongly enhanced pre-osteoblast proliferation and collagen production, while CM-κ-Car most significantly increased alkaline phosphatase activity and mineralization after 28 days. In conclusion, surface-functionalization by SCMC or CM-κ-Car of 3D-printed PCL-scaffolds enhanced pre-osteoblast proliferation and osteogenic activity, likely due to increased surface roughness and hydrophilicity. Surface-functionalization by SCMC most strongly enhanced cell proliferation, while CM-κ-Car most significantly promoted osteogenic activity, suggesting that surface-functionalization by CM-κ-Car may be more promising, especially in the short-term, for *in vivo* bone formation.

## 1 Introduction

Bone tissue engineering requires three-dimensional (3D)-bioactive scaffolds mimicking bone in their structural, chemical, and mechanical properties in order to support cell adhesion, proliferation, and differentiation (Moghaddaszadeh et al., 2021). 3D printing is a favorable technology for producing bone scaffolds with controlled physical and mechanical properties, as well as a hierarchical structure analogous to the bone matrix (Entezari et al., 2019). Poly-ε-caprolactone (PCL) is a commonly used 3D-printable biomaterial (Joseph et al., 2020). It is approved by the United States Food and Drug Administration (FDA) for internal use in the human body, since it is biocompatible and biodegradable, and has a slow degradation rate and favorable mechanical properties (Joseph et al., 2020). Unfortunately, the absence of essential surface features in 3D-printed PCL scaffolds, lacks the inherent mechanical cues for cell attachment, which consequently impairs osteogenesis and *in vivo* integration (Dong et al., 2017). Therefore, it is hypothesized that the bioactivity of these scaffolds can be improved by strategic surface-functionalization.

Numerous surface functionalization techniques including physical (e.g., plasma treatment), chemical (e.g., hydrolysis), or biological (e.g., coating and immobilization of biologically active molecules on the surface) approaches have been implemented to improve the bioactivity of PCL scaffolds (Dwivedi et al., 2020). Glycosaminoglycan (GAG)-like anionic polysaccharides are also widely used for biomaterial functionalization, because of their ability to facilitate cell adhesion, proliferation, and differentiation (Malliappan et al., 2022). Among several kinds of anionic polysaccharides, carboxymethyl cellulose (CMC; a plant-based carboxymethylated polysaccharide) (Seddiqi et al., 2021), and κ-carrageenan (a seaweed-derived sulfated polysaccharide) (Cao et al., 2021) have attracted attention for tissue engineering applications. The ease of chemical functionalization, hydrophilicity, favorable mechanical properties, and biocompatibility make CMC and κ-carrageenan interesting for tissue engineering applications (Cao et al., 2021; Zennifer et al., 2021). The chemical modification of anionic polysaccharides by sulfate and carboxymethyl groups is an important route to enhance their bioactivity (Palhares et al., 2021). The chemical structure of sulfated CMC (SCMC) is similar to heparan sulfate, which facilitates cell adhesion, proliferation, and differentiation (Bhutada et al., 2021). Moreover, it has been shown that carboxymethylated κ-carrageenan (CM-κ-Car) promotes biodegradability, cytocompatibility, stem cell adhesion, cell growth, and osteogenic differentiation (Madruga et al., 2021). However, the effect of SCMC and CM-κ-Car on osteoblast precursor cell adhesion, proliferation, and osteogenic differentiation still needs to be assessed.

In this study, CMC was first sulfated and κ-carrageenan carboxymethylated before their use for surface functionalization of PCL. Then PCL scaffolds were 3D-printed with a regular structure by depositing the strands layer-by-layer with an alternating 0/90° lay-down pattern, since this pattern provides optimal mechanobiological behavior (Saatchi et al., 2020). Afterwards, the effect of surface-functionalization of 3D-printed PCL scaffolds by SCMC or CM-κ-Car was tested experimentally based on the scaffold’s physicochemical and mechanical properties, as well as on the osteogenic response of embedded pre-osteoblasts. Moreover, finite element (FE) modeling was used to quantify the von Mises stress distribution and magnitude under uniform 2% compression strain deformation.

## 2 Materials and methods

### 2.1 Sulfation of carboxymethyl cellulose

**FIGURE 1:**
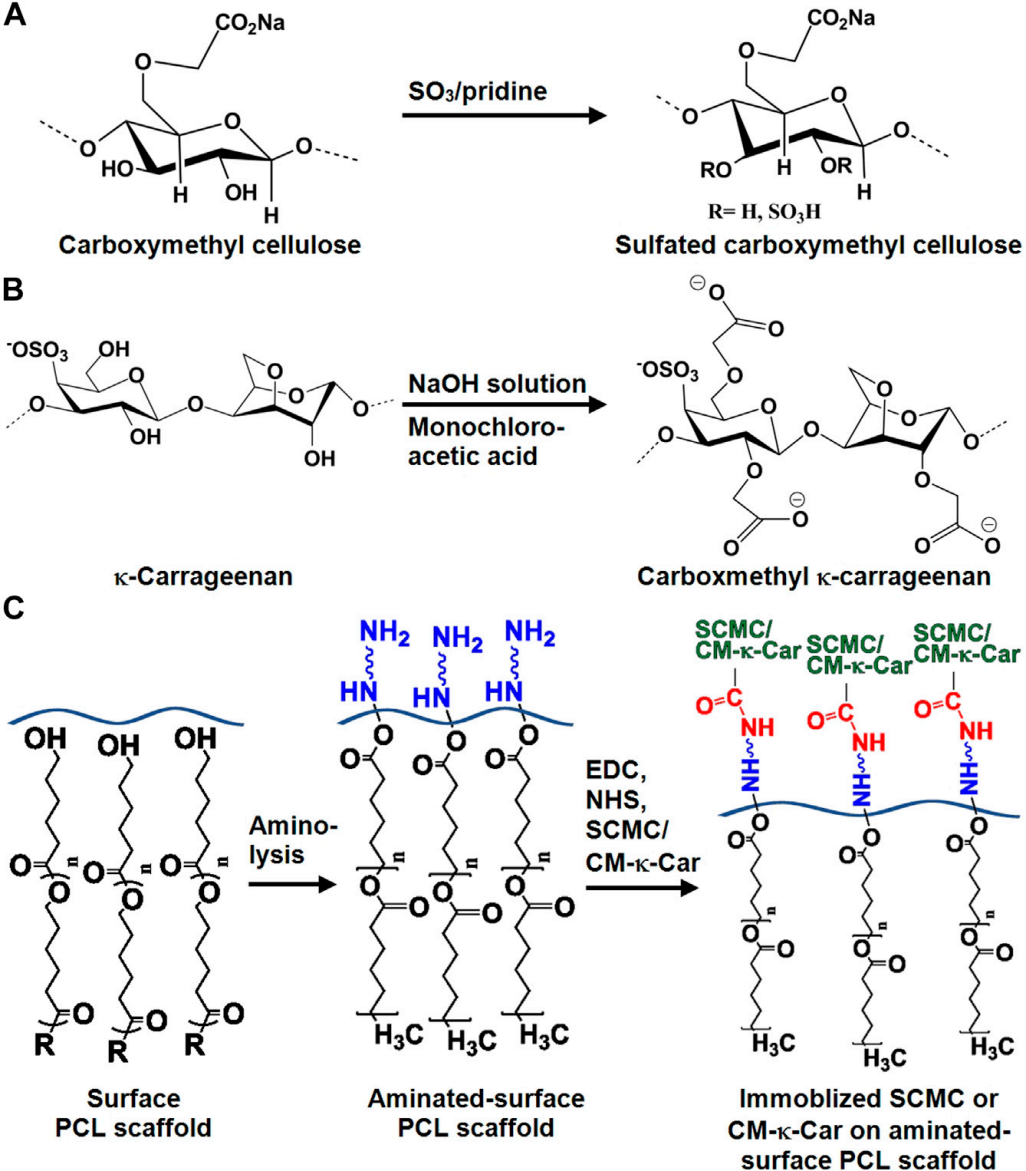
Chemical structure of SCMC and CM-к-Car surface-functionalized 3D-printed PCL scaffolds. **(A)** Chemical conversion of carboxymethyl cellulose to SCMC. **(B)** Chemical conversion of к-carrageenan to CM-к-Car. **(C)** Schematic illustration of surface-functionalization by SCMC or CM-κ-Car of 3D-printed PCL scaffolds. PCL, poly-ε-caprolactone; SCMC, sulfated carboxymethyl cellulose; CM-к-Car, carboxymethyl κ-carrageenan.

CMC was allowed to react with the SO_3_/pyridine complex at room temperature to prepare SCMC as described previously (Hoseinpour et al., 2018). CMC was activated in dimethylacetamide (Merck, Darmstadt, Germany) for 1 h at 80°C while stirring. SO_3_/pyridine complex was prepared through the reaction of pyridine (Merck) and chlorosulfonic acid (Merck). SO_3_/pyridine complex was added to activated CMC, and the reaction was allowed to stir at ambient temperature for 1 h (Figure 1A). The SCMC obtained was dissolved in 0.5 M sodium hydroxide (NaOH; Merck), precipitated in 100% ethanol, and washed in a graded ethanol series [ethanol/water (v/v): 20/80, 40/60, 60/40, 80/20, and 100% ethanol, respectively] to remove remaining salts. Finally, SCMC was dried under vacuum at 50°C.

### 2.2 Carboxymethylation of κ-carrageenan

CM-к-Car was synthesized in two steps as described previously (Ilanlou et al., 2019). Briefly, alkalization of к-carrageenan was achieved using NaOH to form alkoxy-к-carrageenan at 35°C for 1 h under stirring, followed by etherification of alkoxy-к-carrageenan with monochloroacetic acid (Merck, Darmstadt, Germany) for 12 h under stirring to prepare CM-к-Car (Figure 1B). The CM-к-Car (sediment) was frozen at −80°C for 24h, and freeze-dried.

### 2.3 Characterization of sulfated carboxymethyl cellulose and carboxymethyl κ-carrageenan

#### 2.3.1 Fourier transform infrared spectroscopy of sulfated carboxymethyl cellulose and carboxymethyl κ-carrageenan

Fourier transform infrared spectroscopy (FTIR; Thermo Nicolet Avatar 370, San Diego, CA, United States) was used to study the chemical structures of CMC, SCMC, к-carrageenan, and CM-к-Car.

#### 2.3.2 ^1^H and ^13^C nuclear magnetic resonance spectroscopy of sulfated carboxymethyl cellulose and carboxymethyl κ-carrageenan

The ^1^H and ^13^C nuclear magnetic resonance (NMR) spectra of the CMC, SCMC, κ-carrageenan, and CM-κ-Car samples were recorded on a Bruker AMX-500 NMR spectrometer at ambient temperature. The samples were dissolved in D_2_O (35 mg/ml). Chemical shifts (in ppm) were expressed relative to the resonance. MestreNova software (Version 9.0, Mestrelab Research S.L., Santiago de Compostela, Spain) was used to process the data, which included a 90° shifted square sine-bell apodization window. Baseline and phase correction were conducted in both directions.

#### 2.3.3 Degree of substitution of carboxymethyl κ-carrageenan

The degree of substitution (DS) value of CM-к-Car was estimated from potentiometric titration. CM-к-Car (4 g/L) was dissolved in 0.1 M hydrochloric acid (Merck), and titrated with 0.1 M NaOH. The pH values were measured with a pH meter (Mettler Toledo, Columbus, OH, United States) as incremental volumes of NaOH solution were added (Muzzarelli et al., 1984). The DS value was calculated by the following equation:

$$\text{DS}=\left(\text{385×A}\right)/\left(\text{1000}-\text{80}\times \text{A}\right)\text{,A}={\left(\text{V2}-\text{V1}\right)}_{\text{NaOH}}\times {\text{C}}_{\text{NaOH}}{\text{/m}}_{\text{CM-K-Car}}$$ (1)

where A is the amount of–CH_2_COOH and–CH_2_COONa per gram CM-ĸ-Car. Samples were analyzed in triplicate.

### 2.4 Three-dimensional-printing of poly-ε-caprolactone scaffolds

PCL scaffolds (l × w × h:10 mm × 10 mm × 10 mm; volume: 1,000 mm^3^) were printed using a 3DPLN2 bioprinter (3DPL, Tehran, Iran) with a Thermo polymer extruder equipped with a needle (inner diameter: 700 µm). PCL (Mw 80 kDa; Sigma-Aldrich^®^, St. Louis, MO, United States) was heated to 100°C before being extruded at 2 bar through a pre-heated needle. PCL strands were plotted layer-by-layer with an alternating 0°/90° lay-down pattern. In total, 30 scaffolds were printed for this study.

### 2.5 Surface-functionalization of three-dimensional-printed poly-ε-caprolactone scaffolds by sulfated carboxymethyl cellulose or carboxymethyl κ-carrageenan

3D-printed PCL scaffolds were aminolysed with 10% (w/v) 1,6-hexandiamine (Merck, Darmstadt, Germany) in isopropanol (Merck) at 37°C for 1 h, followed by rinsing with deionized water (Figure 1C). For SCMC immobilization, the aminolysed scaffolds were immersed in 1.5% (w/v) SCMC in 0.05 M 2-(N-morpholino) ethanesulfonic acid (pH 6.0; MES; Sigma-Aldrich, St. Louis, MO, United States) buffer solution containing 1.5 mg/ml 1-ethyl-3-(3-dimethylaminopropyl)-carbodiimide (EDC; Sigma-Aldrich) and 1.5 mg/ml N-hydroxysulfosuccinimide (NHS; Thermo Fisher Scientific, Rockville, IL, United States) at ambient temperature for 8 h, followed by rinsing with PBS (Figure 1C). For CM-к-Car immobilization, 2 mg/ml CM-к-Car in 0.5 M MES buffer solution containing 1.6 mg/ml EDS and 3.2 mg/ml NHS was prepared under stirring for 1 h at 37°C. Then aminolysed scaffolds were immersed in CM-к-Car for 4 h at 37°C, rinsed with sodium dihydrogen phosphate (Merck) for 2 h, and rinsed with PBS for 12 h (Figure 1C).

### 2.6 Scaffold characterization

#### 2.6.1 Elemental composition

The elemental composition of the 3D-printed PCL, PCL/SCMC, and PCL/CM-к-Car scaffolds was evaluated with energy dispersive spectroscopy (EDS; Zeiss, Oberkochen, Germany) analysis. Scaffolds were assayed in triplicate.

#### 2.6.2 Hydrophilicity

To determine a possible effect of surface functionalization by SCMC or CM-ĸ-Car on the hydrophilicity of a flat PCL surface prepared by solvent casting, the surface was functionalized by SCMC or CM-ĸ-Car, thereby avoiding possible effects from the curvature and roughness in the scaffolds. The static water contact angle of PCL, PCL/SCMC, and PCL/CM-к-Car sheets was measured according to ASTM D7334 standard (ASTM, 2013). A video contact angle system (Sony color video camera, Tokyo, Japan) was used to capture water contact angle images. The water contact angle was determined using ImageJ. Sheets were analyzed in triplicate.

#### 2.6.3 Surface topography and morphology

The surface topography and morphology of 3D-printed PCL, PCL/SCMC, and PCL/CM-к-Car scaffolds were observed by scanning electron microscopy (SEM). Scaffolds were coated with a layer of gold using an Edwards Sputter Coater S150B (Edwards, Burgess Hill, United Kingdom), and imaged using a Zeiss EVO LS-15 scanning electron microscope (Zeiss, Oberkochen, Germany) with an accelerating voltage of 20 kV.

#### 2.6.4 Void size and strand diameter

Ten voids and strands per scaffold were measured using ImageJ (https://imagej.net/downloads). Scaffolds were assayed in triplicate.

#### 2.6.5 Surface roughness

The surface roughness of 3D-printed PCL, PCL/SCM, and PCL/CM-к-Car scaffolds was determined using a non-contact optical profilometer (Fanavari Kahroba Co., Tehran, Iran). The scaffolds were scanned at 0.5 μm resolution. Data from profilometer were analyzed using Gwyddion (an open-source software package; see gwyddion.net) to determine 3D-scaffold surface topography and average surface roughness. Measurements were performed in triplicate. Scaffolds were analyzed in triplicate.

#### 2.6.6 Surface charge

The zeta potential of the surface of 3D-printed PCL, PCL/SCMC, and PCL/CM-к-Car scaffolds was assessed in 1 mmol/L KCl solution, pH 5.7, at room temperature, using a clamping cell (60 mm × 60 mm) mounted in a SurPASS™3 Electrokinetic Analyzer (Anton Paar GmbH, Graz, Austria), with poly-methyl methacrylate film as reference. Scaffolds were analyzed in triplicate.

#### 2.6.7 Surface chemical composition

Attenuated total reflection-Fourier transform infrared spectroscopy (ATR-FTIR; Thermo Nicolet Avatar 370, San Diego, CA, United States) was used to identify functional groups, chemical interactions, and possible alteration of 3D-printed PCL, PCL/SCMC, and PCL/CM-к-Car scaffolds. Scaffolds were analyzed in triplicate.

#### 2.6.8 Total protein adsorption

To determine total protein adsorption on 3D-printed PCL, PCL/SCMC, and PCL/CM-к-Car scaffolds, the scaffolds were incubated in α-MEM supplemented with 10% FBS at 37°C for 24 h. The scaffolds were then washed with PBS to remove weakly adsorbed proteins and incubated in 2% sodium dodecyl sulfate (Sigma-Aldrich) for 20 h. The total amount of protein was determined by using a BCA Protein Assay reagent Kit (PierceTM, Rockford, lll, United States), and the absorbance was read at 540 nm with a Synergy HT^®^ spectrophotometer (BioTek Instruments, Winooski, VT, United States). Scaffolds were tested in triplicate.

#### 2.6.9 Mechanical properties

3D-printed PCL, PCL/SCMC, and PCL/CM-к-Car scaffolds were tested for their compressive strength using an STM 20 universal testing machine (Santam, Tehran, Iran) with a 200 N load cell at a rate of 1 mm/min at ambient temperature (25°C). Scaffolds were compressed up to 80% of their original length. Stress values were determined by dividing the load by the cross-sectional area of each scaffold (21 mm^2^). Stress-strain curves were plotted, and compressive ultimate strength was assessed. Surface hardness was measured using a Shore durometer (Shore D; Santam; Tehran, Iran). Scaffolds were tested in triplicate.

### 2.7 Finite element modeling

#### 2.7.1 Geometry of the model

A 3D-scaffold (l × w × h: 5 mm × 5 mm × 5 mm) with a strand diameter of 0.5 mm, and a void size of 0.7 mm was designed using commercial software (COMSOL Multiphysics 5.4, Stockholm, Sweden), and used for FE modeling.

#### 2.7.2 Mechanical behavior of the scaffolds

FE modeling was used to measure von Mises stress magnitude and distribution on the 3D-printed PCL scaffolds without or with surface-functionalization, under uniform 2% compression strain. This analysis confirmed no plastic deformation of the scaffolds under the conditions mentioned. The 3D structure of the scaffolds was modeled by considering the scaffold as a homogenous and isotropic linear elastic material. The bottom surface of the scaffolds was fixed, and a displacement-controlled boundary condition was applied to the top surface of the scaffolds. The final displacement was set as an equivalent vertical strain of 2%, which was within the range of elastic deformation for this composite material (Baptista and Guedes, 2020). The models were meshed using 208,779 tetrahedra, 116,514 triangles, 29,652 edges, and 5220 vertex elements. Table 1 provides detailed information on the model parameters used for modeling.

**TABLE 1:** Parameters and default values used in the FE modeling.

Type of scaffold	Parameter	Expression	Value	References
PCL	E (MPa)	Elastic modulus	162.2	
	ϑ	Poisson’s ratio	0.3	(Liu et al., 2020)
PCL/SCMC	E (MPa)	Elastic modulus	147.5	
	ϑ	Poisson’s ratio	0.3	
PCL/CM-к-Car	E (MPa)	Elastic modulus	119.5	
	ϑ	Poisson’s ratio	0.3	

PCL, poly-ε-caprolactone; SCMC, sulfated carboxymethyl cellulose; CM-к-Car, carboxymethyl κ-carrageenan.

The computed reaction force (F_R_) defines the scaffold’s effective elastic modulus (E) according to Eq. 2:

$$\text{E}={\text{F}}_{\text{R}}/\left(\text{A}\times \text{ɛ}\right)$$ (2)

where the average strain *ε* = 0.02, and the cross-sectional area of the compression A = 21 mm^2^.

### 2.8 Cell culture and pre-osteoblast bioactivity

#### 2.8.1 Cell culture, and seeding onto the scaffolds

MC3T3-E1 pre-osteoblasts (American Type Culture Collection, Manassas, VA, United States) were grown and maintained in α-Minimum Essential Medium (α-MEM; Gibco, Life Technologies, Walthan, MA, United States), supplemented with 10% fetal bovine serum (FBS; Gibco, Life Technologies, Waltham, MA, United States), and 1% PSF (antibiotic antimycotic solution, Sigma-Aldrich^®^, St. Louis, MO, United States), in a humidified incubator with 5% CO_2_ in air at 37°C. After reaching 75% confluency, cells were detached using 0.25% trypsin (Gibco, Invitrogen, Waltham, MA, United States) and 0.1% ethylenediaminetetraacetic acid (Merck, Darmstadt, Germany) in PBS at 37°C. Cells were then resuspended at 5 × 10^6^ cells/ml in osteogenic medium consisting of α-MEM supplemented with 10% FBS, 1% PSF, 50 μg/ml ascorbic acid (Sigma-Aldrich^®^, St. Louis, MO, United States), and 10 mM β-glycerophosphate (Sigma-Aldrich^®^, St. Louis, MO, United States).

Cell seeding was performed by spreading ten 10 µl drops of a 5 × 10^5^ cells/cm^3^ cell suspension onto the scaffold surface in 24-well culture plates. Cell-seeded scaffolds were incubated for 3 h at 5% CO_2_ in the air at 37°C to allow cell attachment. To prevent evaporation, 100 µl osteogenic medium was added every 30 min during this period. Then osteogenic medium (1,500 µl/well) was added, and cell-seeded scaffolds were cultured for up to 28 days. The culture medium was changed every 2 days. Cell seeding efficiency, morphology, spreading, gene expression, and proliferation were assessed as described below. At days 7, 14, 21, and 28, scaffolds were collected and cut into 8 equal parts (l × w × h: 5 mm × 5 mm × 5 mm; volume: 125 mm^3^), since cell distribution from the scaffold’s top to bottom was relatively homogeneous. Each part of all types of scaffold was compared with respect to collagen production, ALP activity, and matrix mineralization (see below). For comparison between groups, parts from the same location in the scaffolds were used (Zamani et al., 2018).

#### 2.8.2 Cell morphology and spreading

Cell morphology and spreading on 3D-printed PCL, PCL/SCMC, and PCL/CM-к-Car scaffolds were observed an imaged by SEM after 4 h, 12 h, and 3 days of culture. Cell-seeded scaffolds were fixed using 4% glutaraldehyde, followed by dehydration in a graded ethanol series (50, 70, 80, 90, and 100%). Cell-seeded scaffolds were coated with a layer of gold using an Edwards Sputter Coater S150B, and imaged using a Zeiss EVO LS-15 scanning electron microscope with an accelerating voltage of 20 kV.

#### 2.8.3 Cell seeding efficiency

To determine seeding efficiency, cell-seeded scaffolds were kept under static conditions for 8 h following cell seeding to guarantee sufficient time for cell attachment and adaptation to a specific scaffold architecture. The cell-seeded scaffolds, in a 24-well culture plate (“old plate”), were washed twice with PBS, and transferred to a new 24-well culture plate. Seeding efficiency was assessed by determining the number of cells attached to the wells of the “old plate” as well as the number of cells attached to the scaffolds, using AlamarBlue^®^ fluorescent assay (Invitrogen, Frederick, MD, United States), according to the manufacturer’s instructions. We determined a linear relationship between AlamarBlue^®^ fluorescence and cell number (data not shown). Fresh osteogenic medium with 10% AlamarBlue^®^ was added to the wells of the “old plate” and each scaffold until the solution completely covered the top of the scaffolds. Both scaffolds and “old plate” were incubated in AlamarBlue^®^ solution for 4 h in a humidified incubator with 5% CO_2_ at 37°C. The solution was harvested from the scaffolds and the “old plate”, and the fluorescence was measured at 530 nm with a Synergy HT^®^ spectrophotometer. Scaffolds were washed twice with PBS to remove AlamarBlue^®^, and incubated in a humidified incubator with 5% CO_2_ in air at 37°C. Seeding efficiency was calculated according to the following equation:

$$\text{Seeding efficiency }\left(\text{\%}\right)=\frac{number\;of\;cells\;attached\;to\;scaffold\;}{number\;of\;cells\;attached\;to\;scaffold\;+\;number\;of\;cells\;attached\;to\;plate\;}\times 100$$ (3)

Three independent experiments with 3 constructs per group were assayed in triplicate.

#### 2.8.4 Analysis of gene expression

After 1 or 7 days of culture, total RNA was extracted using TRIzol^®^ reagent (Life Technologies, Waltham, MA, United States), and stored at -80°C prior to analysis. Complementary DNA (cDNA) was synthesized using the First Strand cDNA Synthesis kit (Thermo Fisher Scientific, Vilnius, Lithuania). cDNA was stored at −20°C prior to real-time-polymerase chain reaction (RT-PCR) analysis, and diluted 5× for gene expression analysis. RT-PCR reactions were performed using 1 μl cDNA per reaction and LightCycler^®^ 480 SYBR^®^ Green I Mastermix (Roche Diagnostics, Mannheim, Germany) in a LightCycler^®^ 480 (Roche Diagnostics). RT-PCR conditions for all genes were as follows: 10 min pre-incubation at 95°C, followed by 45 cycles of amplification at 95°C for 10 s, 56°C for 5 s, 72°C for 10 s, and 78°C for 5 s, after which melting curve analysis was performed. With LightCycler^®^ software (version 1.2), crossing points were assessed and plotted versus the serial dilution of known concentrations of the internal standard (Jin et al., 2021). Target proliferation marker gene *Ki67* and osteogenic marker genes *Runx2*, *Cox2, Ocn, Fgf2,* and *Dmp1* were analyzed. Gene expression levels were calculated relative to the housekeeping gene *Pbgd*. Primer sequences are listed in Table 2. Three independent experiments with five scaffolds were performed.

**TABLE 2:** Primers used in real time PCR.

Gene	Forward primer sequence (5′-3′)	Reverse primer sequence (5′-3′)
*Runx2*	ATGCTTCATTCGCCTCAC	ACT​GCT​TGC​AGC​CTT​AAA​T
*Ocn*	CAG​ACA​CCA​TGA​GGA​CCA​TCT​T	GGT​CTG​ATA​GCT​CGT​CAC​AA
*Ki67*	CCC​TCA​GCA​AGC​CTG​AGA​A	AGA​GGC​GTA​TTA​GGA​GGC​AAG
*Fgf2*	GGC​TTC​TTC​CTG​CGC​ATC​CA	TCC​GTG​ACC​GGT​AAG​TAT​TG
*Cox2*	TTG​CTG​TTC​CAA​TCC​ATG​TCA	GGT​GGG​CTT​CAG​CAG​TAA​TTT​G
*Dmp1*	CGG​CTG​GTG​GAC​TCT​CTA​AG	CGG​GGT​CGT​CGC​TCT​GCA​TC
*Pbgd*	AGT​GAT​GAA​AGA​TGG​GCA​ACT	TCT​GGA​CCA​TCT​TCT​TGC​TGA

*Runx2,* runt-related transcription factor 2; *Ocn,* osteocalcin; *Ki67,* antigen KI-67; *Fgf2,* fibroblast growth factor-2; *Cox2,* cyclooxygenase-2; *Dmp1,* dentin matrix acidic phosphoprotein 1; *Pbgd,* porphobilinogen deaminase.

#### 2.8.5 Cell proliferation

MC3T3-E1 pre-osteoblast proliferation was assessed by determining the cell number in the scaffolds at days 3, 7, 14, 21, and 28, and by dividing these numbers by the cell number in the scaffolds at day 1, using AlamarBlue^®^ fluorescent assay, as described above, using Eq. 1. At each time point, scaffolds were transferred to a new plate, AlamarBlue^®^ was added, and the fluorescence was measured. After performing the AlamarBlue^®^ assay on each day, scaffolds were washed twice with PBS, and incubated in osteogenic medium in a humidified incubator with 5% CO_2_ at 37°C. Data was obtained from three scaffolds from three independent experiments (*n* = 3).

#### 2.8.6 Alkaline phosphatase activity and protein assay

Alkaline phosphatase (ALP) activity was measured to determine the osteoblastic phenotype of MC3T3-E1 pre-osteoblasts on 3D-printed PCL, PCL/SCMC, and PCL/CM-к-Car scaffolds. On days 7, 14, 21, and 28 of cell culture on the scaffolds, one part (1/8th) of the cell/scaffold construct was subjected to cell lysis. Cells were lysed with milli-Q water, and freeze-thawed 3 times to determine ALP activity and protein content. P-nitrophenyl-phosphate (Merck, Darmstadt, Germany) at pH 10.3 was used as the substrate for ALP as described earlier (Kroeze et al., 2011). The absorbance was read at 410 nm. ALP activity was expressed as µmol/µg total cellular protein. The amount of protein was determined by using a BCA Protein Assay reagent Kit (Pierce™, Rockford, lll, United States), and the absorbance was read at 540 nm with a Synergy HT^®^ spectrophotometer (BioTek Instruments, Winooski, VT, United States). Constructs were assayed in triplicate.

ALP protein was stained after 7, 14, 21, and 28 days of culture. One part (1/8th) of the cell/scaffold construct was washed 3 times with PBS, and fixed with 4% formaldehyde in PBS for 15 min at 37°C. The BCIP/NBT (5-bromo-4-chloro-3-indolyl phosphate (BCIP)/nitro blue tetrazolium (NBT) phosphatase color development kit (Roche Diagnostics, Mannheim, Germany) was used for the colorimetric detection of ALP intensity by incubation for 30 min at 37°C. Optical images were taken using a stereomicroscope.

#### 2.8.7 Collagen production

Total collagen production by MC3T3-E1 pre-osteoblasts on 3D-printed scaffolds was visualized and quantified using a picrosirius red stain kit (Chondrex, Inc., Redmond, WA, United States). After 7, 14, 21, and 28 days of culture, one part (1/8th) of the cell/scaffold construct was washed with PBS thrice and fixed in 4% formaldehyde. Fixed constructs were stained for 2 h with picrosirius red at room temperature. Then, constructs were washed twice with acidified water (5 ml acetic acid/L distilled water) to remove the unbound stain, and collagen production was visualized using a Nikon SMZ-10 stereomicroscope (Nikon, Tokyo, Japan) and a Leica inverted microscope (Leica Microsystems, Wetzlar, Germany). For semiquantitative collagen analysis, picrosirius red stain was eluted from the constructs using 0.2 M NaOH/methanol (1:1, v/v) for 30 min under shaking. A 100 μl of this solution per well of a 96-well plate (Greiner, Bio-One, Alphen aan den Rijn, Netherlands) was used to determine the absorbance at 490 nm, with a microplate reader (BioRad Laboratories Inc., Veenendaal, Netherlands). Constructs were weighed after 24 h of air drying at room temperature. Data were normalized to the weight of the dried construct and expressed as absorbance/g. Constructs were assayed in triplicate.

#### 2.8.8 Matrix mineralization

ECM mineralization by MC3T3-E1 pre-osteoblasts attached to the scaffolds was analyzed after 7, 14, 21, and 28 days of culture. To determine mineralization, one part (1/8th) of the cell/scaffold construct was washed with PBS, and fixed in 4% glutaraldehyde for 15 min at 37°C. Fixed constructs were incubated in 40 mM Alizarin Red staining solution (Merck, Darmstadt, Germany), pH 4.3, at room temperature for 30 min, and washed extensively with deionized water to remove the unreacted dye. Optical images were taken using a stereomicroscope. For quantitative mineralization analysis, the red-stained mineralized nodules were dissolved with 5% sodium dodecyl sulfate (Merck) in 0.5 N HCL at room temperature under shaking. Then, 100 µl of the solution was added per well of a 96-well plate (Techno Plastic Products, Trasadingen, Switzerland) to measure the absorbance at 405 nm with a microplate reader (BioRad Laboratories Inc., Veenendaal, Netherlands). Scaffolds were air-dried at room temperature for 24 h and weighed. The absorbance values were normalized to the weight of the scaffolds and expressed as absorbance/g. Constructs were assayed in triplicate.

### 2.9 Statistical analysis

All data are expressed as mean ± standard deviation (SD) from at least three independent, separate experiments. Data were analyzed using one-way ANOVA, and the significance of differences among means of contact angle, elastic modulus, comprehensive strength, surface roughness, strand diameter, void size, zeta potential, surface hardness, and protein adsorption were determined by post-hoc comparisons, using Bonferroni’s method. Two-way analysis of variance with pairwise comparison was used to assess differences among means of marker gene expression, pre-osteoblast proliferation, ALP activity, collagen production, and matrix mineralization between groups and over time. Differences were considered significant if *p* < 0.05. Statistical analysis was performed using GraphPad Prism^®^ 8.0 (GraphPad Software Inc.).

## 3 Results

### 3.1 Chemical structure and composition of sulfated carboxymethyl cellulose and carboxymethyl κ-carrageenan

**FIGURE 2:**
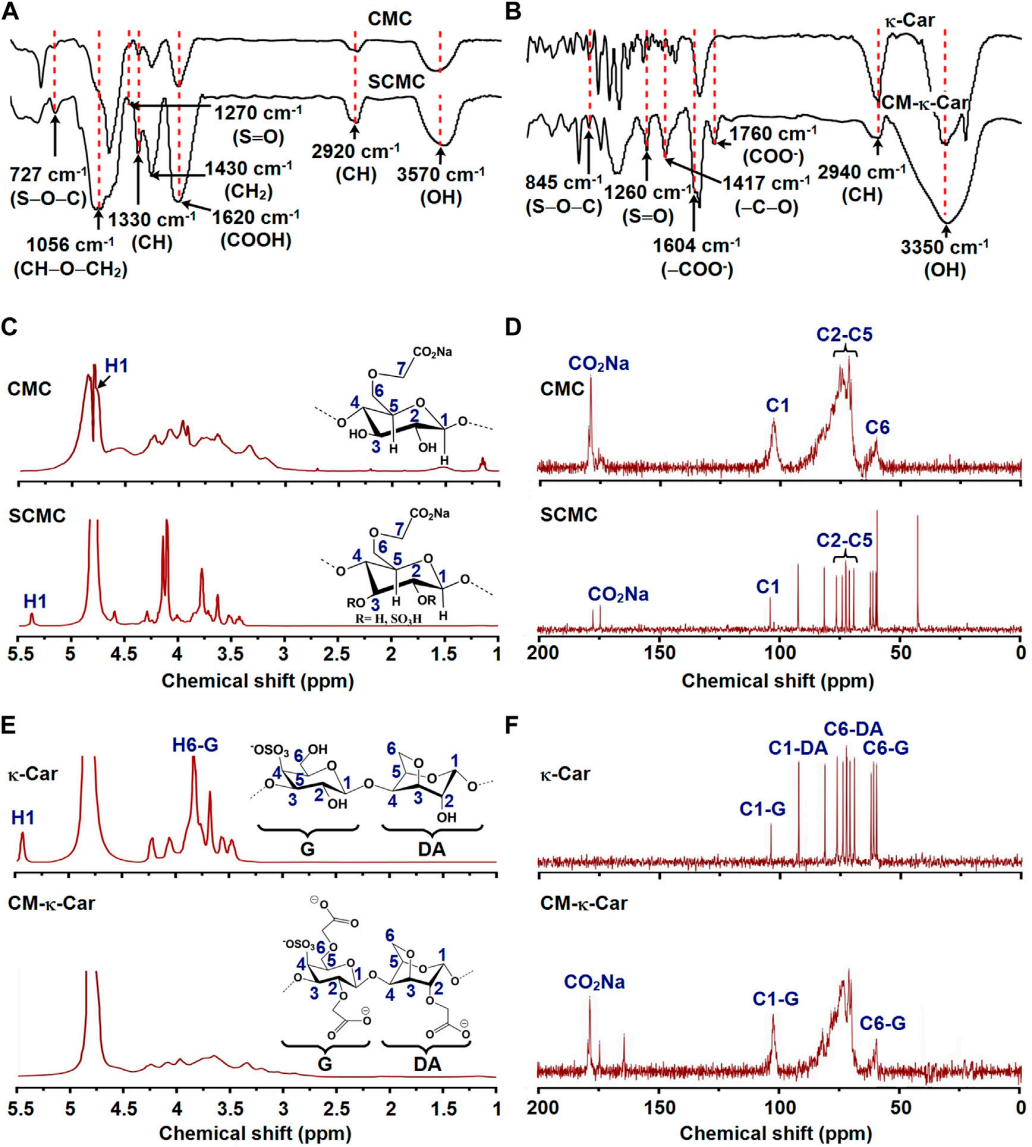
Effect of functionalization of CMC by sulfate groups and κ-Car by carboxymethyl groups on chemical composition and structure. **(A)** FTIR spectra of CMC and SCMC. **(B)** FTIR spectra of κ-Car and CM-κ-Car. FTIR, Fourier-transform infrared spectroscopy; CMC, carboxymethyl cellulose; SCMC, sulfated carboxymethyl cellulose; к-Car, κ-carrageenan; CM-к-Car, carboxymethyl κ-carrageenan. **(C)** ^1^H NMR spectra of CMC and SCMC. **(D)** ^13^C NMR spectra of CMC and SCMC. **(E)** ^1^H NMR spectra of κ-Car and CM-κ-Car. **(F)** ^13^C NMR spectra of κ-Car and CM-κ-Car. NMR, Nuclear magnetic resonance; CMC, carboxymethyl cellulose; SCMC, sulfated carboxymethyl cellulose; к-Car, κ-carrageenan; CM-к-Car, carboxymethyl κ-carrageenan.

The chemical structure and composition of CMC, SCMC, κ-Car, and CM-κ-Car were determined by FTIR spectroscopy, and ^1^H NMR and ^13^C NMR spectroscopy (Figure 2)*.*

#### 3.1.1 Fourier transform infrared spectroscopy spectra of sulfated carboxymethyl cellulose and carboxymethyl κ-carrageenan

CMC characteristic peaks were detected at 3,570 (OH stretching), 2,920 (asymmetric CH stretching), 1,620 (COOH stretching), 1,430 (CH_2_ stretching), 1,330 (CH stretching), and 1,056 (CH‒O‒CH_2_ stretching) (Bhutada et al., 2021). After sulfation of CMC, two new bonds appeared at 1,270 and 727 cm^−1^ in the SCMC spectra, corresponding to the stretching vibration bands of S═O and S‒O‒C (Figure 2A) (Bhutada et al., 2021). In the spectra of κ-Car, characteristic peaks were detected at 3,350 (OH stretching), 2,940 (asymmetric CH stretching), 1,260 (S═O stretching), and 845 cm^−1^ (S‒O‒C stretching) (Mobarak et al., 2012). After carboxymethylation of κ-Car, new characteristic peaks appeared at 1,604 and 1760 cm^−1^ in CM-κ-Car spectra corresponding to the stretching vibration band of ‒COO^−^, indicating carboxymethylation of κ-Car (Figure 2B) (Mobarak et al., 2012; Ilanlou et al., 2019). In addition, the increase in the intensity of the absorption peak at 1,417 cm^−1^ (symmetric ‒C‒O stretching) confirmed carboxymethylation of κ-Car (Mobarak et al., 2012).

#### 3.1.2 ^1^H nuclear magnetic resonance and ^13^C nuclear magnetic resonance spectra of sulfated carboxymethyl cellulose and carboxymethyl κ-carrageenan

The chemical structures of CMC, SCMC, κ-Car, and CM-κ-Car were studied by ^1^H NMR and ^13^C NMR spectra (Figure 2). The ^1^H NMR spectrum of CMC revealed the expected signals of 7 protons (H1-H7) (Figure 2C). The signals of H2–H7 were shown as a group of overlapping peaks between 3.17 and 4.23 ppm (Figure 2C) (Bhutada et al., 2021). The ^1^H NMR spectrum of SCMC revealed downfield displacement of CMC proton signals due to the addition of electron-withdrawing sulfate (SO_4_^−^) groups (Figure 2C) (Bhutada et al., 2021). The signal of H1 (anomeric proton) of CMC (4.76 ppm) shifted downfield (5.36 ppm), which could be ascribed to de-shielding of the C2 sulfate group (Figure 2C). The chemical shifts of C2–C5 in CMC were between 71.26 and 75.01 ppm (Figure 2D) (Fan et al., 2014). After sulfation, the C2 and C3 shifted to lower field positions, since they were directly attached to electronegative sulfate ester groups. The C4 and C5 shifted to higher field positions, since they were indirectly attached to sulfate ester groups (Figure 2D) (Fan et al., 2014).

The ^1^H NMR spectrum of κ-carrageenan and CM-κ-Car revealed peaks ranging between 3 and 5 ppm (Figure 2E) (Mobarak et al., 2012). The existence of new peaks around 3.17–3.35 confirmed the substitution of a carboxymethyl group to a hydroxyl group in κ-carrageenan (Figure 2E) (Mobarak et al., 2012). The presence of a new peak at 178 ppm was attributed to a carboxylated carbon in CM-κ-Car, which confirmed the carboxylation of κ-carrageenan (Figure 2F) (Mobarak et al., 2012). The DS value of CM-κ-Car was 0.92 ± 0.004 [Values are mean ± SD (*n* = 3)].

### 3.2 Surface chemistry, hydrophilicity, and topography

**FIGURE 3:**
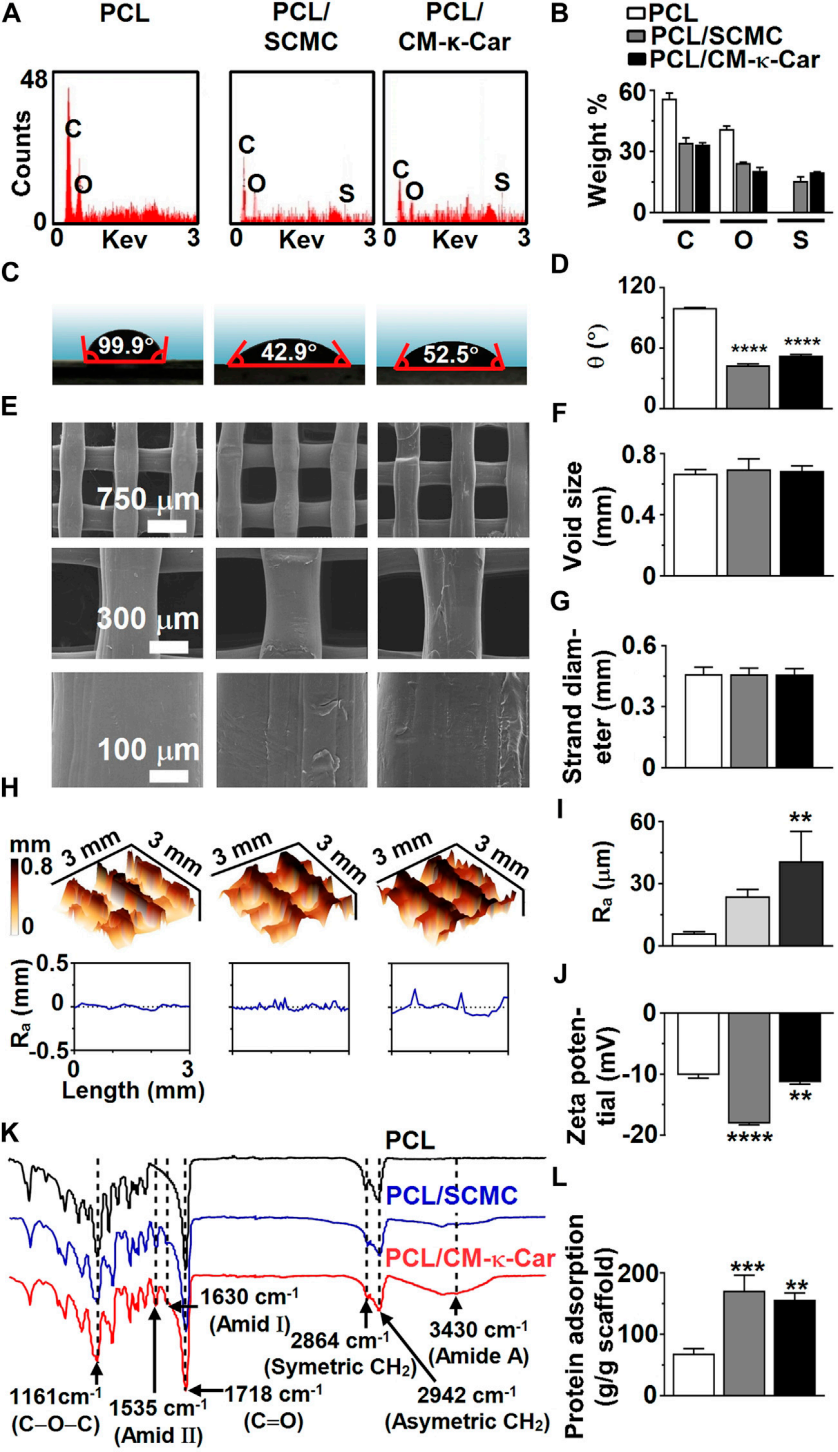
Effect of surface-functionalization of 3D-printed PCL scaffolds by SCMC or CM-κ-Car on surface chemistry, hydrophilicity, and topography. **(A)** EDX spectra indicating chemical elements. **(B)** Weight percent of carbon, oxygen, nitrogen, and sulfate. **(C)** Water contact angle on surface of scaffolds. **(D)** Average water contact angle. **(E)** SEM images showing the structure, morphology, and surface topography of the strands inside the scaffolds. **(F)** Void size. **(G)** Strand diameter. **(H)** 3D-view of scaffold surface topography showing surface-functionalization-dependent variation in surface roughness distribution and magnitude. *R*_a_, average surface roughness. **(I)** Average surface roughness. **(J)** Zeta potential. **(K)** FTIR spectra indicating functional groups. **(L)** Protein adsorption on surface of scaffolds. Values are mean ± SD (*n* = 3). ***Significantly different from unfunctionalized 3D-printed PCL scaffold, *p* < 0.0005; ****p < 0.0001. PCL, poly-ε-caprolactone; SCMC, sulfated carboxymethyl cellulose; CM-к-Car, carboxymethyl κ-carrageenan; EDX, energy dispersive X-ray spectroscopy; FTIR, Fourier-transform infrared spectroscopy, C, carbon; O, oxygen; N, nitrogen; S, sulfate. *Significantly different from unfunctionalized 3D-printed PCL scaffold, *p* < 0.05, ****p < 0.0001. PCL, poly-ε-caprolactone; SCMC, sulfated carboxymethyl cellulose; CM-к-Car, carboxymethyl κ-carrageenan; SEM, scanning electron microscopy.

The elemental composition of 3D-printed PCL scaffolds with or without surface functionalization was analyzed by EDX spectral analysis (Figures 3A,B). EDX spectra of 3D-printed PCL scaffolds indicated the presence of carbon [C; 55.95 ± 1.95 wt% (mean ± SD)], and oxygen (O; 41.05 ± 1.49 wt%) (Figure 3B). EDX spectra of 3D-printed PCL/SCMC scaffolds indicated the presence of carbon (C; 34.20 ± 1.80 wt%), oxygen (O; 24.35 ± 0.35 wt%), and sulfur (S; 15.50 ± 1.98 wt%) (Figure 3B). EDX spectra of 3D-printed PCL/CM-κ-Car scaffolds indicated the presence of carbon (C; 33.35 ± 0.65 wt%), oxygen (O; 20.45 ± 1.63 wt%), and sulfur (S; 19.75 ± 0.21 wt%) (Figure 3B). Surface-functionalization by SCMC or CM-κ-Car was validated by the increment in the percentage of sulfur on PCL/SCMC and PCL/CM-κ-Car scaffolds (Figure 3B). Surface-functionalization by CM-κ-Car created higher sulfate groups (1.27-fold) on the surface of 3D-printed PCL scaffolds compared to SCMC (Figure 3B).

The hydrophilicity of the 3D-printed PCL scaffolds was analyzed by water contact angle measurement (Figures 3C,D). Surface-functionalization by SCMC and CM-κ-Car did affect the hydrophilicity of scaffolds (Figure 3C). The water contact angle was lower on PCL/SCMC (0.43-fold, *p* < 0.0001) and PCL/CM-κ-Car (0.53-fold, *p* < 0.0001) scaffolds than on unfunctionalized PCL scaffolds (Figure 3D). In addition, the water contact angle was lower on PCL/SCMC scaffolds (0.82-fold) than on PCL/CM-κ-Car scaffolds (Figure 3D).

Unfunctionalized 3D-printed PCL scaffolds had a regular structure with a smooth surface (Figure 3E). Surface-functionalization of PCL scaffolds by SCMC or CM-κ-Car resulted in surface irregularity with a surface topology exhibiting small peaks and troughs (Figure 3E). Surface-functionalization by SCMC or CM-κ-Car did not change the void size and strand diameter of PCL scaffolds (Figures 3F,G). Surface-functionalization by SCMC and CM-κ-Car had an effect on the PCL scaffolds’ surface roughness distribution and magnitude (Figure 3H). The average surface roughness was higher on PCL/CM-κ-Car (6.62-fold, *p* < 0.005), but not PCL/SCMC, than on unfunctionalized PCL scaffolds (Figure 3I). The zeta potential was measured to determine the (negative) surface charge of the PCL (−10.14 ± 0.50 mV), PCL/SCMC (−18.09 ± 0.22 mV), and PCL/CM-κ-Car (−11.34 ± 0.30 mV) scaffolds (Figure 3J). The surface charge was negatively higher on PCL/SCMC scaffolds (1.78-fold, *p* < 0.0001) and PCL/CM-κ-Car scaffolds (1.12-fold, *p* < 0.005) than on unfunctionalized PCL scaffolds (Figure 3J).

The FTIR spectra of unfunctionalized 3D-printed PCL scaffolds showed PCL characteristic peaks at 2,942 cm^−1^ [asymmetric (CH_2_) stretching], 2,864 cm^−1^ [symmetric (CH_2_) stretching], 1718 cm^−1^ [carbonyl (C=O) group stretching], and 1,161 cm^−1^ [ester bond (C–O–C) stretching] (Figure 3K) (Zamani et al., 2018). The FTIR spectra of surface-functionalized scaffolds by SCMC and CM-κ-Car showed characteristic peaks at wavelengths 3,430 (amid A), 1,630 (amid Ι), and 1,535 cm^−1^ (amid ΙΙ; Figure 3K) (Ilanlou et al., 2019; Alamry and Khan, 2021).

Surface-functionalization by SCMC and CM-κ-Car did affect protein adsorption on 3D-printed PCL scaffolds (Figure 3L). Protein adsorption was higher on PCL/SCMC (2.49-fold, *p* < 0.0005) and PCL/CM-κ-Car (2.28-fold, *p* < 0.005) scaffolds than on unfunctionalized PCL scaffolds (Figure 3L).

### 3.3 Mechanical properties

**FIGURE 4:**
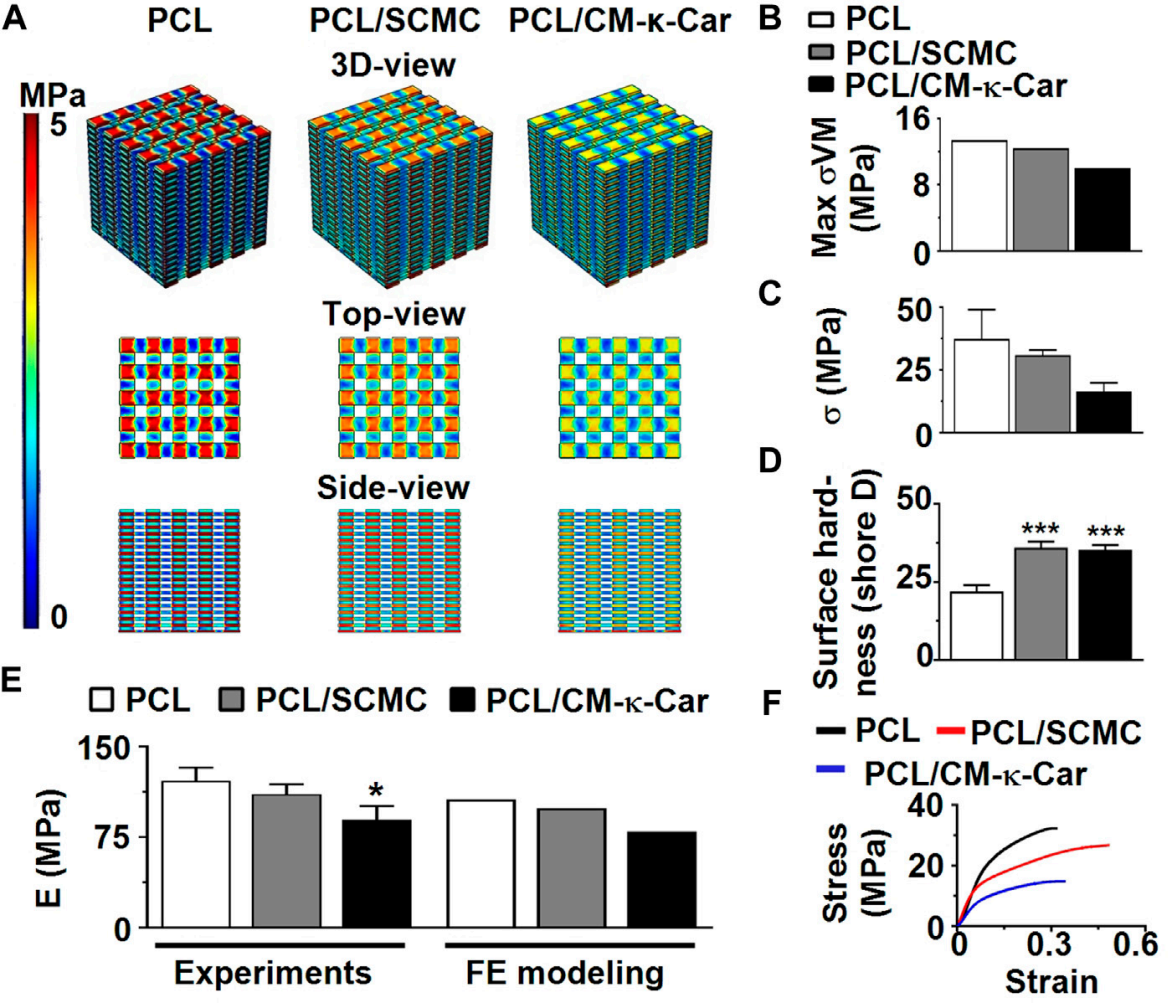
Effect of 3D-printed PCL scaffold surface-functionalization by SCMC or CM-κ-Car on mechanical properties. **(A)** 3D-view, top-view, and side-view of von Mises stress distribution on the scaffolds as a result of uniform 2% compression strain deformation determined by FE modeling. **(B)** Maximum von Mises stress. **(C)** Compressive strength. **(D)** Surface hardness. **(E)** Elastic modulus determined experimentally and by FE modeling. **(F)** Stress-strain curve. Values are mean ± SD (*n* = 3). *Significantly different from unfunctionalized 3D-printed PCL scaffold, *p* < 0.05, ****p < 0.0001. PCL, poly-ε-caprolactone; SCMC, sulfated carboxymethyl cellulose; CM-к-Car, carboxymethyl κ-carrageenan; FE, finite element modeling.

The von Mises stress distribution was less homogeneous on unfunctionalized PCL scaffolds than on PCL/SCMC and PCL/CM-κ-Car scaffolds (Figure 4A). The maximum von Mises stress for 2% compression strain ranged between 10.1 and 13.4 MPa for all types of scaffolds (Figure 4B). In all types of scaffolds, the maximal von Mises stress did not exceed the yield stress of bulk materials (Figure 4B). The surface-functionalization by SCMC and CM-κ-Car on PCL scaffolds did not significantly change the compressive strength (Figure 4C). The surface hardness was higher on PCL/SCMC (1.64-fold, *p* < 0.0005) and PCL/CM-κ-Car scaffolds (PCL/CM-κ-Car: 1.61-fold, *p* < 0.0005) compared to unfunctionalized PCL scaffolds (Figure 4D). The elastic modulus was lower on PCL/CM-κ-Car scaffolds (0.74-fold, *p* < 0.05), but not PCL/SCMC scaffolds, compared to unfunctionalized PCL scaffolds (Figure 4E). The variation in mean values of FE modeling and experimental results was only 8%–14%, which is generally accepted in validating FE modeling results by experimental data (Figure 4E) (Gupta et al., 2004). The surface-functionalization by SCMC and CM-κ-Car did not change the stress-strain relationship (Figure 4F).

### 3.4 Pre-osteoblast spreading, attachment, and gene expression

**FIGURE 5:**
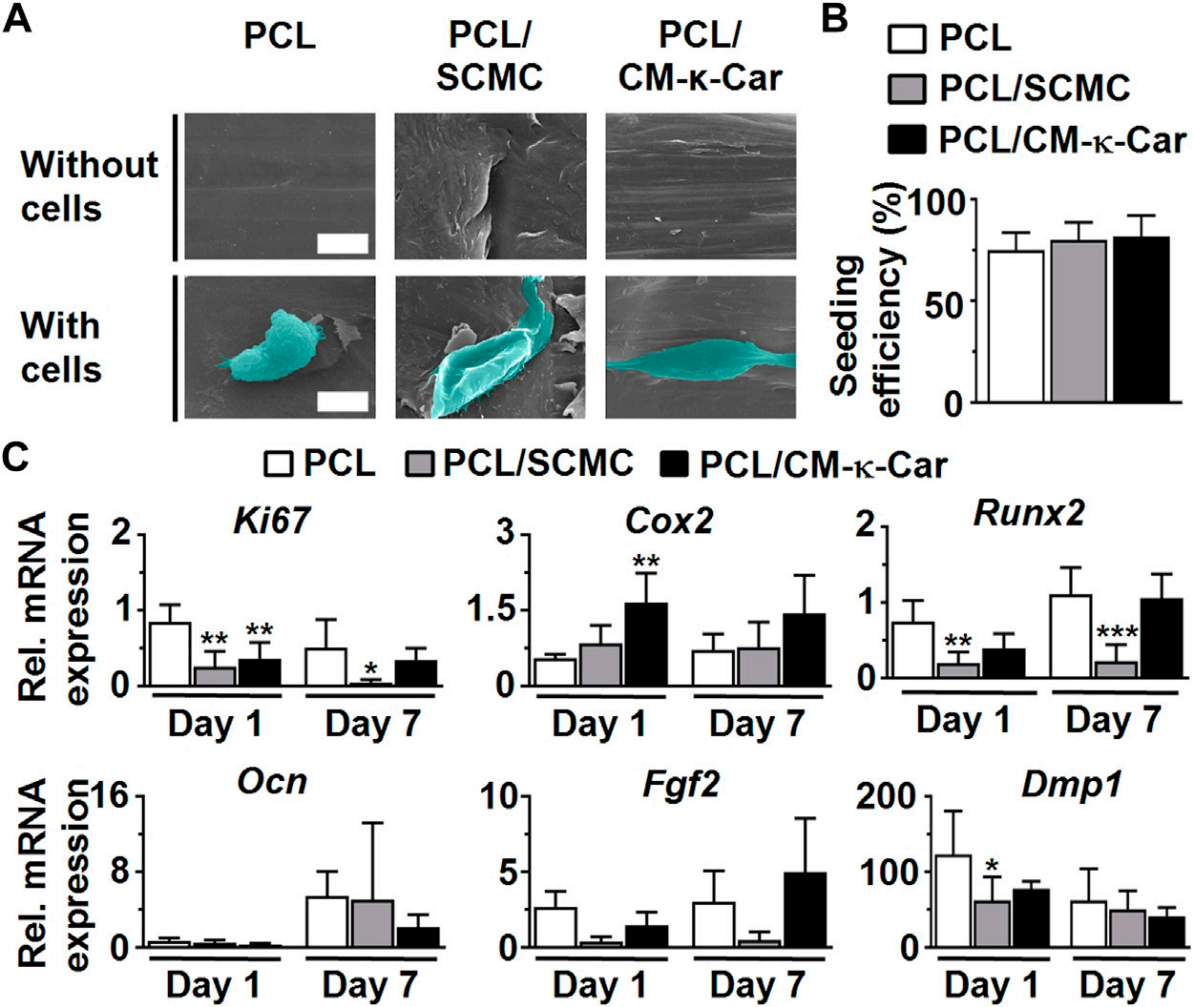
Effect of 3D-printed PCL scaffold surface-functionalization by SCMC or CM-κ-Car on MC3T3-E1 pre-osteoblast spreading, attachment, and gene expression. **(A)** SEM images showing pre-osteoblast morphology and spreading after 4 h. Scale bar: 10 µm. **(B)** Pre-osteoblast seeding efficiency. **(C)** Expression of osteogenic (*Runx2*, *Ocn*, *Dmp1*), proliferation (*Ki67*), and angiogenic-related (*Fgf2*) genes by MC3T3-E1 pre-osteoblasts after 1 and 7 days. Values are normalized to *Pbgd* expression. Values are mean ± SD (*n* = 3). *Significantly different from unfunctionalized 3D-printed PCL scaffolds, *p* < 0.05, ***p < 0.0005, ****p < 0.0001. PCL, poly-ε-caprolactone; SCMC, sulfated carboxymethyl cellulose; CM-к-Car, carboxymethyl κ-carrageenan; SEM, scanning electron microscopy.

Surface-functionalization by SCMC and CM-κ-Car had an effect on pre-osteoblast morphology and spreading, as visualized by SEM imaging, on 3D-printed PCL scaffolds after 4 h of culture (Figure 5A). Pre-osteoblasts did not spread well on the surface of unfunctionalized PCL scaffolds and exhibited a slightly spherical morphology (Figure 5A). Surface-functionalization by SCMC and CM-κ-Car improved cell spreading on 3D-printed PCL scaffolds (Figure 5A). Well-spread cells with a natural spindle-shaped morphology were observed on the surface of PCL/SCMC and PCL/CM-κ-Car scaffolds (Figure 5A). Surface-functionalization by SCMC and CM-κ-Car did not change the pre-osteoblast seeding efficiency on 3D-printed PCL scaffolds after 8 h (Figure 5B). Seeding efficiency ranged between 75% (on PCL scaffolds) and 82% (on PCL/CM-κ-Car scaffolds) (Figure 5B).

Surface-functionalization by SCMC and CM-κ-Car did affect the expression of a proliferation marker gene and osteogenesis-related genes in pre-osteoblasts cultured on 3D-printed PCL scaffolds after 1 and 7 days (Figure 5C). *Ki67* gene expression was higher on 3D-printed PCL scaffolds compared to SCMC (0.30-fold, *p* < 0.005) and CM-κ-Car (0.42-fold, *p* < 0.005) surface-functionalized scaffolds after 1 day, but after 7 days, only PCL/SCMC (0.09-fold, *p* < 0.05) exhibited lower *Ki67* gene expression compared to PCL scaffolds. *Cox2* mRNA levels were higher on PCL/CM-κ-Car scaffolds (3.00-fold, *p* < 0.005), but not on PCL/SCMC scaffolds, compared to unfunctionalized PCL scaffolds after 1 day (Figure 5C). *Cox2* mRNA levels were not significantly different on all 3D-printed PCL scaffolds, with or without surface-functionalization at day 7 (Figure 5C). *Runx2* mRNA levels were higher on PCL/SCMC scaffolds, but not on PCL/CM-κ-Car scaffolds, after 1 day (0.26-fold, *p* < 0.005) or after 7 days (0.20-fold, *p* < 0.0005) compared with unfunctionalized PCL scaffolds (Figure 5C). There were no significant differences in *Ocn* and *Fgf2* gene expression between 3D-printed PCL scaffolds with or without surface-functionalization after 1 or 7 days (Figure 5C). *Dmp1* mRNA levels were lower on PCL/SCMC scaffolds (0.51-fold, *p* < 0.05), but not on PCL/CM-κ-Car scaffolds, compared to unfunctionalized PCL scaffolds after 1 day (Figure 5C). However, *Dmp1* mRNA levels did not change on 3D-printed PCL scaffolds with or without surface-functionalization after 7 days (Figure 5C).

### 3.6 Pre-osteoblast proliferation

**FIGURE 6:**
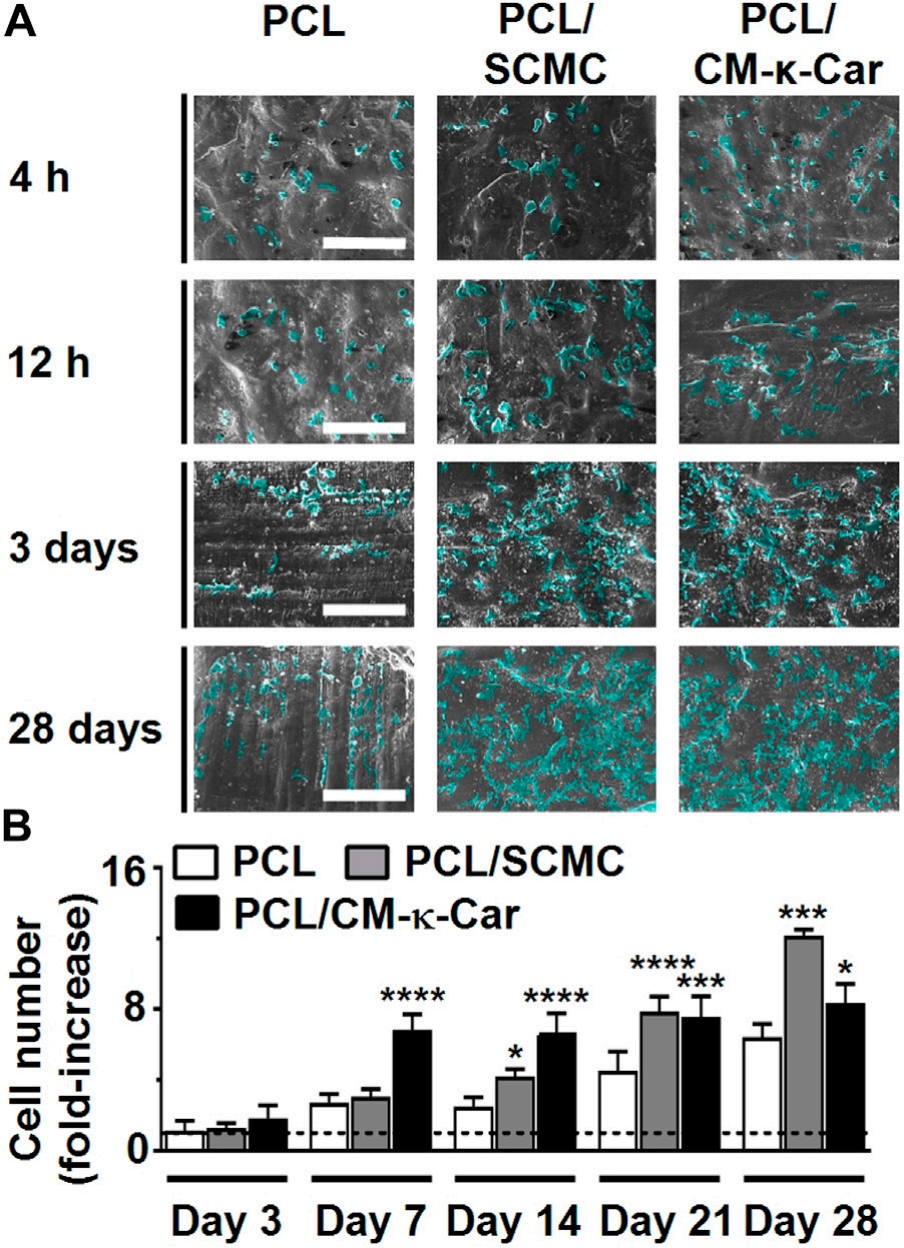
Effect of 3D-printed PCL scaffold surface-functionalization by SCMC or CM-κ-Car on MC3T3-E1 pre-osteoblast proliferation. **(A)** SEM images showing pre-osteoblasts after 4 h, 12 h, 3 days, and 28 days of culture. Scale bar: 200 µm. **(B)** Quantification of pre-osteoblast proliferation after 3, 7, 14, 21, and 28 days relative to day 1. Values are mean ± SD (*n* = 3). *Significantly different from unfunctionalized 3D-printed PCL scaffolds, *p* < 0.05, **p < 0.005, ****p < 0.0001. PCL, poly-ε-caprolactone; SCMC, sulfated carboxymethyl cellulose; CM-к-Car, carboxymethyl κ-carrageenan; SEM, scanning electron microscopy.

Surface-functionalization by SCMC and CM-κ-Car affected pre-osteoblast spreading and proliferation as visualized by SEM imaging, on 3D-printed PCL scaffolds after 4 h, 12 h, 3 days, and 28 days of culture (Figure 6A). Well-spread and proliferating cells were observed on the surface of PCL/SCMC and PCL/CM-κ-Car scaffolds (Figure 6A). Surface-functionalization by SCMC or CM-κ-Car did change pre-osteoblast proliferation on 3D-printed PCL scaffolds after 7, 14, 21, and 28 days in relation to day 1 (Figure 6B). At day 3, pre-osteoblast proliferation was similar on all types of scaffolds, while at day 7, pre-osteoblast proliferation was significantly higher on PCL/CM-κ-Car scaffolds (2.50-fold, *p* < 0.0001), but not on the PCL/SCMC scaffolds, than on unfunctionalized PCL scaffolds (Figure 6B). At day 14, pre-osteoblast proliferation was significantly higher on PCL/SCMC scaffolds (1.67-fold, *p* < 0.05) and on PCL/CM-κ-Car scaffolds (2.66-fold, *p* < 0.0001) than on unfunctionalized PCL scaffolds (Figure 6B). After 21 days, cell proliferation was higher on PCL/SCMC (1.73-fold, *p* < 0.0001) and PCL/CM-κ-Car (1.66-fold, *p* < 0.0005) scaffolds, and after 28 days, when cell proliferation was also higher on PCL/SCMC (1.89-fold, *p* < 0.0005) and on PCL/CM-κ-Car (1.30-fold, *p* < 0.05) scaffolds than on unfunctionalized scaffolds (Figure 6B).

### 3.7 Alkaline phosphatase activity and protein

**FIGURE 7:**
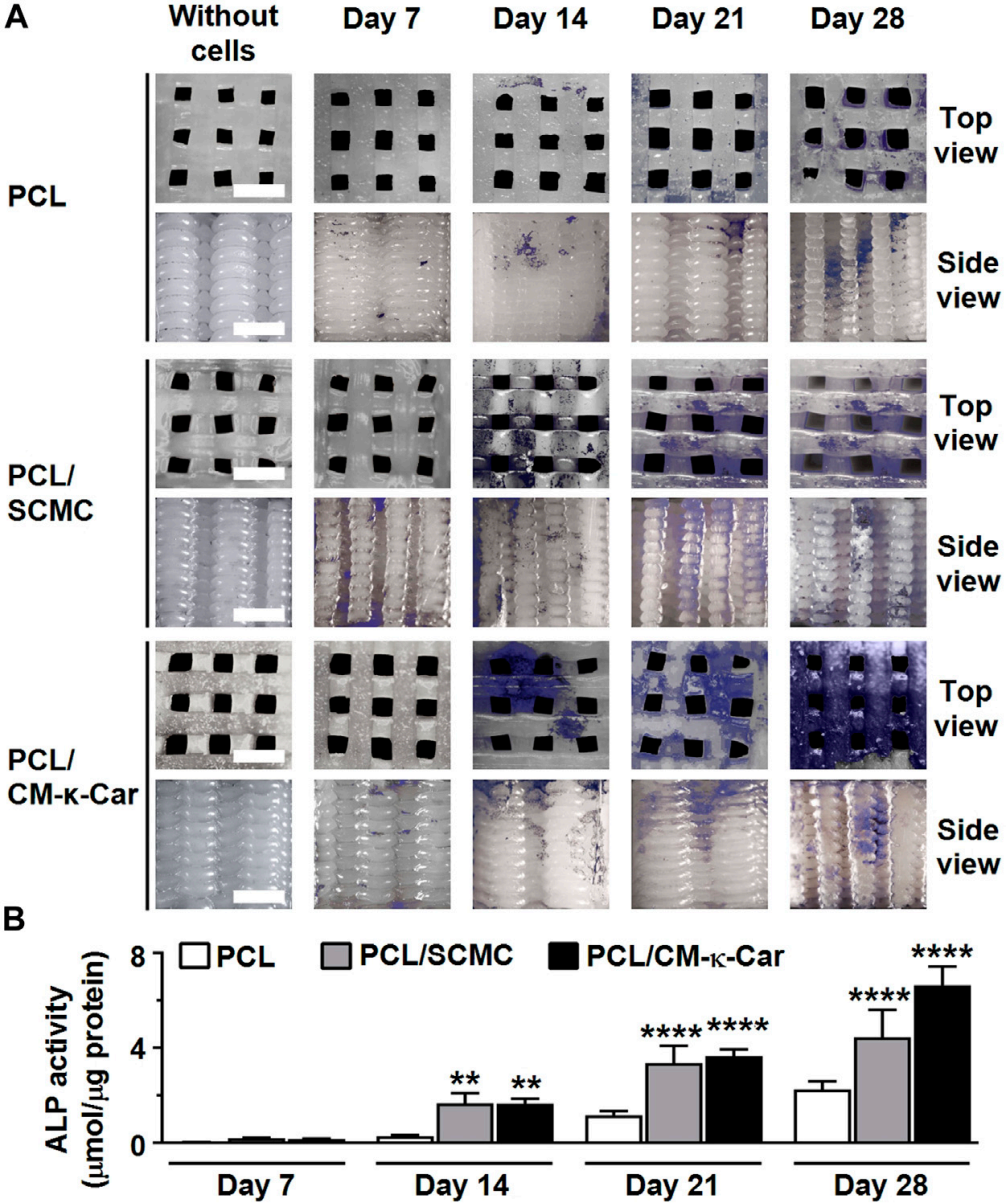
Effect of 3D-printed PCL scaffold surface-functionalization by SCMC or CM-κ-Car on ALP activity of MC3T3-E1 pre-osteoblasts after 7, 14, 21, and 28 days. **(A)** ALP staining (purple). Scale bar: 1.5 mm. **(B)** Quantification of ALP activity. Values are mean ± SD (*n* = 3). **Significantly different from control 3D-printed PCL scaffolds, *p* < 0.005, ****p < 0.0001. PCL, poly-ε-caprolactone; SCMC, sulfated carboxymethyl cellulose; CM-к-Car, carboxymethyl κ-carrageenan.

The osteoinductive ability of the scaffolds was evaluated by measuring ALP activity. ALP activity (blue) was increased, i.e., more intense blue staining, on PCL/CM-κ-Car scaffolds after 14, 21, and 28 days of culture compared to all other scaffolds (Figure 7A). At day 7, ALP activity was similar on all types of scaffolds (Figure 7B). At day 14, ALP activity was higher in PCL/SCMC (6.05-fold, *p* < 0.005) and on PCL/CM-κ-Car (6.02-fold, *p* < 0.005) scaffolds than on unfunctionalized scaffolds. Also, at day 21, ALP activity was higher on PCL/SCMC (2.90-fold, *p* < 0.0001) and on PCL/CM-κ-Car (3.14-fold, *p* < 0.0001) scaffolds than on unfunctionalized scaffolds (Figure 7B). Finally, at day 28, ALP activity was higher on PCL/SCMC (1.97-fold, *p* < 0.0001) and on PCL/CM-κ-Car (2.94-fold, *p* < 0.0001) scaffolds than on unfunctionalized PCL scaffolds (Figure 7B).

### 3.8 Collagenous matrix production

**FIGURE 8:**
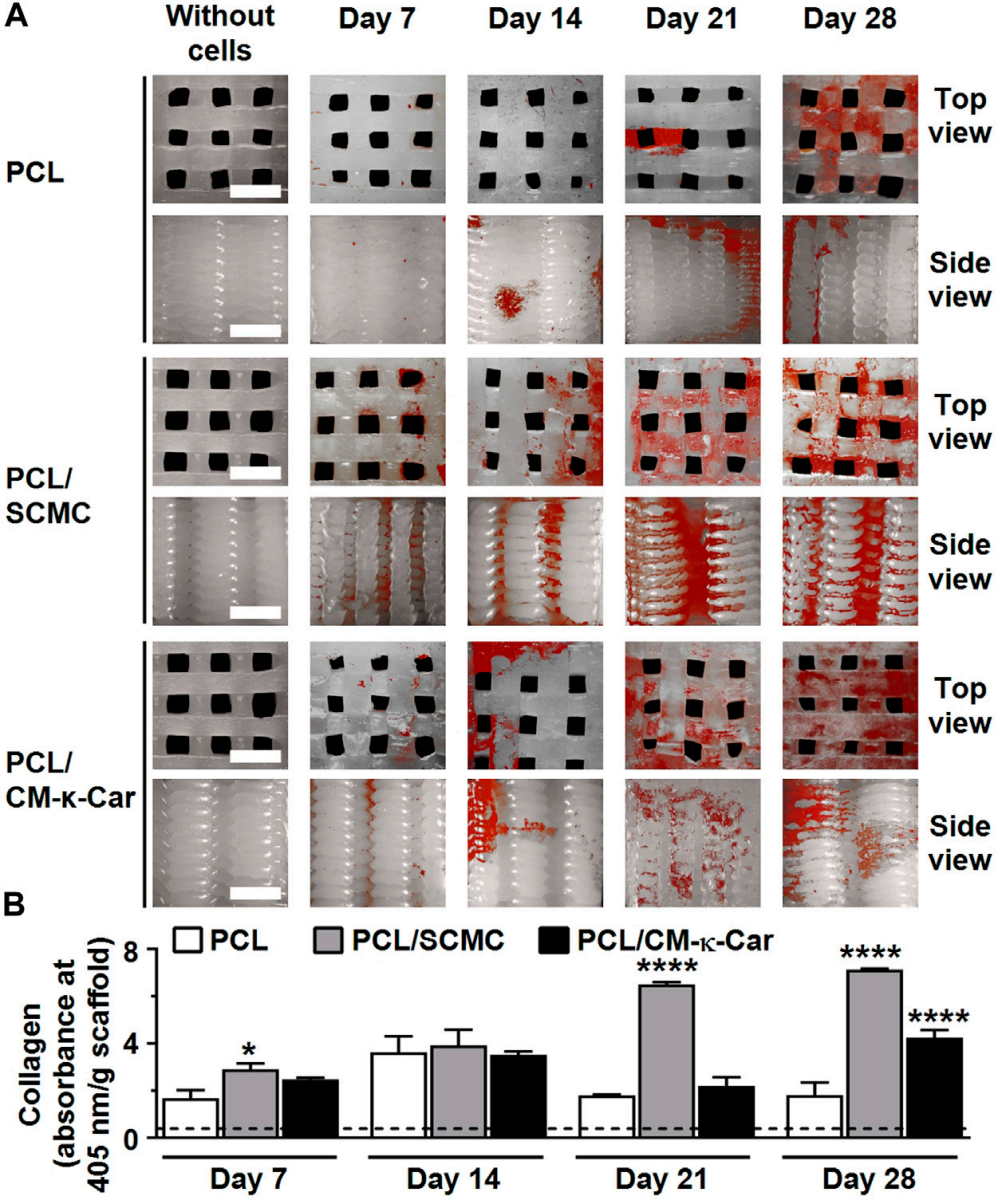
Effect of 3D-printed PCL scaffold surface-functionalization by SCMC or CM-κ-Car on collagen production by MC3T3-E1 pre-osteoblasts after 7, 14, 21, and 28 days. **(A)** Collagen staining (red). Collagenous matrix production, visualized by picrosirius red staining. Scale bar: 1.5 mm. **(B)** Quantification of collagen production. Values are mean ± SD (*n* = 3). *Significantly different from control 3D-printed PCL scaffolds, *p* < 0.05, ****p < 0.0001. PCL, poly-ε-caprolactone; SCMC, sulfated carboxymethyl cellulose; CM-к-Car, carboxymethyl к-carrageenan.

Collagen production (red) was enhanced, i.e., more intense red staining, on PCL/SCMC scaffolds after 7, 14, 21, and 28 days of culture compared to all other scaffolds (Figure 8A). At day 7, collagen production was higher on PCL/SCMC (1.73-fold, *p* < 0.05) scaffolds, but not on PCL/CM-κ-Car scaffolds, than on unfunctionalized PCL scaffolds (Figure 8B). At day 14, collagen production was similar on all types of scaffolds (Figure 8B). At day 21, collagen production was higher on PCL/SCMC (3.62-fold, *p* < 0.0001) scaffolds, but not on PCL/CM-κ-Car scaffolds, than on unfunctionalized PCL scaffolds (Figure 8B). At day 28, collagen production was higher on PCL/SCMC (3.95-fold, *p* < 0.0001) and on PCL/CM-κ-Car (2.35-fold, *p* < 0.0001) scaffolds than on unfunctionalized PCL scaffolds (Figure 8B).

### 3.9 Matrix mineralization

**FIGURE 9:**
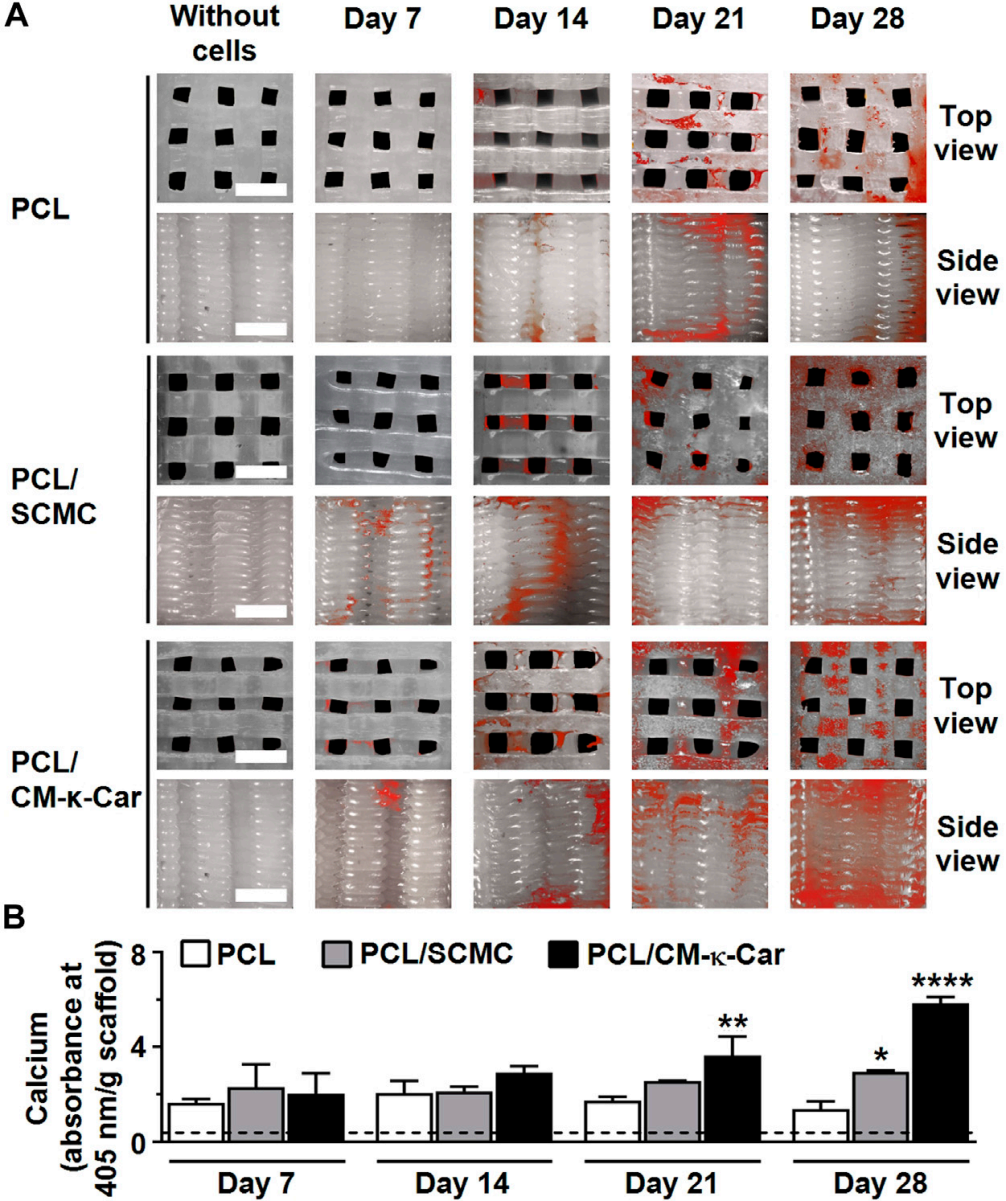
Effect of 3D-printed PCL scaffold surface-functionalization by SCMC or CM-κ-Car on matrix mineralization by MC3T3-E1 pre-osteoblasts after 7, 14, 21, and 28 days. **(A)** Mineral deposition staining (alizarin red). Scale bar: 1.5 mm. **(B)** Quantified mineral content. Values are mean ± SD (*n* = 3). *Significantly different from control 3D-printed PCL scaffolds, *p* < 0.05, **p < 0.005, ****p < 0.0001. PCL, polycaprolactone; SCMC, sulfated carboxymethyl cellulose; CM-к-Car, carboxymethyl κ-carrageenan.

Surface-functionalization by SCMC and CM-κ-Car did affect matrix mineralization on 3D-printed PCL scaffolds after 7, 14, 21, and 28 days (Figure 9A). Matrix mineralization (red) was increased, i.e., more intense red staining, on PCL/CM-κ-Car scaffolds after 28 days of culture compared to all other scaffolds (Figure 9A). At days 7 and 14, matrix mineralization was similar on all types of scaffolds (Figure 9B). At day 21, matrix mineralization was higher on PCL/CM-κ-Car (2.11-fold, *p* < 0.005) scaffolds, but not on PCL/SCMC scaffolds, than on unfunctionalized PCL scaffolds (Figure 9B). At day 28, mineralization was higher on PCL/SCMC (2.12-fold, *p* < 0.05) and PCL/CM-κ-Car (4.23-fold, *p* < 0.0001) scaffolds than on unfunctionalized PCL scaffolds (Figure 9B).

## 4 Discussion

PCL is widely used to fabricate 3D-printed scaffolds. However, cell attachment and/or proliferation are not supported by PCL, resulting from a lack of bioactivity (Park et al., 2021). Surface-functionalization by bioactive agents can significantly improve the biological properties of 3D-printed PCL scaffolds (Park et al., 2021). Anionic polysaccharides, like CMC and κ-carrageenan, are used as naturally-derived bioactive agents for bone tissue engineering, based on their bone-like biological properties to facilitate cell adhesion, proliferation, and differentiation (Singh et al., 2016; Goonoo et al., 2017). The bioactivity of CMC and κ-carrageenan can be effectively increased by chemical modification, e.g., by sulfation and/or carboxymethylation (Madruga et al., 2020; Bhutada et al., 2021). The current study aimed to investigate the effects of surface-functionalization by SCMC or CM-κ-Car on the physicochemical and mechanical properties of 3D-printed PCL scaffolds, as well as the osteogenic response of pre-osteoblasts. We hypothesized that surface-functionalization by SCMC or CM-κ-Car on a 3D-printed PCL scaffold significantly affects the scaffold’s physicochemical and mechanical properties, as well as the osteogenic response of pre-osteoblasts. We found that 1) Surface-functionalization by SCMC or CM-κ-Car did not change the scaffold geometry and structure; 2) Surface-functionalization by SCMC or CM-κ-Car similarly increased surface charge, hydrophilicity, surface roughness, and hardness; 3) Using FE-modeling, on all types of scaffolds the maximal von Mises stress for 2% compression strain did not exceed the yield stress for the bulk-material, showing that the scaffold will not show irreversible deformation when in use; 4) Surface-functionalization by SCMC, but not CM-κ-Car, decreased *Runx2* and *Dmp1* expression, while surface-functionalization by CM-κ-Car, but not by SCMC, increased *Cox2* expression; and 5) Surface-functionalization by SCMC most strongly enhanced proliferation and collagen production, while CM-κ-Car most significantly increased ALP activity and mineralization. Thus, our results revealed increased osteogenic differentiation potential in PCL/CM-κ-Car scaffolds compared to PCL/SCMC scaffolds *in vitro*, suggesting that surface-functionalization by CM-κ-Car may be more promising, especially in the short-term, for *in vivo* bone formation.

Our data confirmed that sulfation of CMC and carboxymethylation of k-carrageenan successfully occurred, suggesting improved CMC and k-carrageenan bioactivity (Hoseinpour et al., 2018; Madruga et al., 2020). Sulfation of carboxymethyl cellulose increases ALP activity and expression of osteogenic genes, e.g., osterix and noggin (Peschel et al., 2012). Carboxymethylation of k-carrageenan increases cytocompatibility, biodegradability, cell adhesion and growth, and osteogenic differentiation of stem cells (Madruga et al., 2021).

The surface chemical composition of scaffolds regulates osteoblast function and differentiation (Zreiqat et al., 2005). We found that the oxygen concentration on the surface of the strands in the PCL/SCMC scaffolds was higher compared to PCL/CM-κ-Car scaffolds, indicating the presence of more carboxymethyl groups in the PCL/SCMC scaffolds compared to PCL/CM-κ-Car scaffolds. Since carboxymethylation of poly (2-hydroxyethyl methacrylate) pellets has been shown to enhance osteoblast adherence and proliferation (Filmon et al., 2002), our results suggest that PCL/SCMC scaffolds are more favorable than PCL/CM-κ-Car scaffolds for pre-osteoblast attachment and proliferation. We also found that the sulfur concentration on the surface of the strands in the PCL/CM-κ-Car scaffolds was higher compared to PCL/SCMC scaffolds, illustrating more sulfate groups in the PCL/CM-κ-Car scaffolds compared to PCL/SCMC scaffolds. Since sulfate groups promote osteogenic differentiation (Ruiz Velasco et al., 2011), our findings suggest that PCL/CM-κ-Car scaffolds stimulate osteogenic differentiation more strongly than PCL/SCMC scaffolds.

Our data showed that surface functionalization of PCL scaffolds by SCMC or CM-κ-Car introduced polar molecules, *i.e.* oxygen and sulfur, at the surface, resulting in a more negatively charged scaffold surface. A negative surface charge improves osteogenic activity in Saos-2 and MC3T3-E1 cells (Radha et al., 2021). Therefore, a negative surface charge, as we found in our study, might suggest enhanced osteogenic activity as a result of SCMC or CM-κ-Car surface-functionalization.

Our data showed that 3D-printed PCL scaffolds had a regular structure and interconnected pores. Such a structure and porosity promote oxygen diffusion and cell proliferation (Seddiqi et al., 2020). As expected, surface-functionalization by SCMC and CM-κ-Car did not change the scaffolds’ structure. Moreover, we found that the average void size of the surface-functionalized scaffolds ranged from 600 to 700 µm. Such a void size promotes cell growth and matrix mineralization *in vitro*, as well as bone formation *in vivo* (Ouyang et al., 2019). We also found that surface-functionalization by SCMC or CM-κ-Car resulted in surface irregularity with peaks and troughs, indicating that our surface functionalization affected protein adsorption and cell behavior, as has been shown for PCL films (Khampieng et al., 2018). Surface roughness, on a micron scale, improves cell attachment, viability, and osteogenic differentiation (Zhang et al., 2015; Lukaszewska-Kuska et al., 2018). We showed that surface-functionalization by SCMC or CM-κ-Car of PCL scaffolds changed surface roughness distribution, and slightly increased surface roughness. Surface-functionalization by CM-κ-Car most significantly increased surface roughness. Therefore, these surface-functionalized scaffolds might promote cell attachment, viability, and osteogenic differentiation *in vitro*, as has been observed for surface-modified titanium with increased surface roughness (Lukaszewska-Kuska et al., 2018).

Cells can only adhere to the surface of a biomaterial after protein from body fluid is adsorbed onto the biomaterial. Our data showed that surface functionalization of PCL scaffolds by SCMC or CM-κ-Car improves protein adsorption. This was expected since surface hydrophilicity and roughness enhance protein adsorption (Akkas et al., 2013). In addition, a negative surface charge aids protein adsorption through ionic and hydrogen bonding (Aramesh et al., 2015). The presence of sulfate groups on the scaffold surface also improves protein adsorption (Kim et al., 2010). Adsorbed proteins, such as fibronectin and vitronectin, activate the focal adhesion pathway, ECM-receptor interaction pathway, and regulate actin cytoskeleton formation (Yang et al., 2013). Enhanced fibronectin adsorption on a sulfated polymer-grafted surface stimulates the pre-osteoblast attachment (Felgueiras et al., 2015). Fibronectin also plays an important role in cell proliferation and differentiation (Linsley et al., 2013). Moreover, it has been shown that sulfated polymer grafting onto a scaffold surface increases the secretion of fibronectin and collagen by osteoblasts (Pavon-Djavid et al., 2007). The sulfated polysaccharide ĸ-Car promotes paxillin protein expression by pre-osteoblasts (Cao et al., 2021). Protein adsorption on a scaffold surface promotes cell adhesion, proliferation, and osteogenic differentiation (Gandavarapu et al., 2013). Therefore, our finding that cell attachment, proliferation and osteogenic activity were increased on surface-functionalized PCL scaffolds by SCMC or CM-κ-Car might be related to improved protein adsorption.

3D-printed scaffolds should have a favorable mechanical strength to meet the mechanical requirements for target bone tissue and should retain their structure to maintain their mechanical function after implantation (Zamani et al., 2020). Our data showed that surface functionalization of PCL scaffolds by SCMC or CM-κ-Car did not meaningfully change the elastic modulus and compressive strength. Note that the scaffolds were not meant to deform beyond the elastic, reversible region, and the ultimate compressive strength was controlled, as a safety feature. Therefore, the reduction in ultimate compressive strength had little importance to the functionality of the scaffolds. Furthermore, we showed that for all types of scaffolds the maximal von Mises stress for 2% compression strain was within the elastic region. We also showed that the surface hardness of the surface-functionalized scaffolds was remarkedly increased, which provided a more bone-like feature to promote osteogenic differentiation due to the stiffer surface, thus modulating mechano-transduction pathways (Shih et al., 2011).

Cell attachment and spreading are necessary steps toward the clinical translation of 3D scaffolds to be used for tissue engineering applications (Sobral et al., 2011). We found that the seeding efficiency of all surface-functionalized and unfunctionalized 3D-printed PCL scaffolds was similar, likely because the same large 3D-scaffold structure enabled cell entrapment. Moreover, we found that pre-osteoblasts exhibited a slightly spherical morphology on unfunctionalized 3D-printed PCL scaffolds. This was expected since PCL is hydrophobic and lacks biological recognition sites (Park et al., 2021). In addition, we found that pre-osteoblasts exhibited a well-spread morphology on scaffolds that were surface-functionalized by SCMC or CM-κ-Car compared to PCL scaffolds. This can be explained by increased hydrophilicity, as well as by the presence of carboxyl and sulfate groups in the chemical structures of SCMC and CM-κ-Car.

Cell-material interaction is crucial for cell survival and cellular functions such as cell viability, proliferation, differentiation, and mineralization (Zamani et al., 2018). In our study, SCMC or CM-κ-Car surface-functionalized 3D-printed PCL scaffolds did not affect *Ki67* gene expression by pre-osteoblasts at day 1 and 7. Since proliferation was enhanced by SCMC or CM-κ-Car surface-functionalization, *Ki67* gene expression might be changed at a later time point. Surface-functionalization of all 3D-printed scaffolds did not affect *Ocn* and *Fgf2* gene expression at day 1 and 7, but did affect *Dmp1* expression at day 1. These three genes are known to be related to extracellular matrix (ECM) mineralization in the long term, and therefore their expression might not be affected significantly immediately upon exposure to a surface-functionalized PCL scaffold, but only at later time points. We found that 3D-printed PCL scaffold surface functionalization by SCMC or CM-κ-Car considerably affected the scaffold’s physicochemical properties, which might have affected ECM mineralization *via* expression of other molecules, e.g., E11/gp38 glycoprotein or sclerostin (Prideaux et al., 2012). Future studies are needed to test this hypothesis. *Cox2* and *Runx2* gene expression were upregulated respectively downregulated by SCMC and CM-κ-Car surface functionalization. The downregulation of *Runx2* gene expression was unexpected and needs further experimentation for clarification. The SCMC or CM-κ-Car surface functionalization-stimulated osteogenic differentiation might mainly rely on *Cox2* activity in the short-term (before day 7). Based on the long-term data (ALP activity, collagen production, and matrix mineralization), other osteogenic genes were likely involved in osteogenic differentiation of pre-osteoblasts cultured on 3D-printed PCL scaffolds with or without surface functionalization.

3D scaffolds must promote cell proliferation inside their structures. Our data showed that cell proliferation was similar in all types of scaffolds after 3 days, but diverged after 7 days. SCMC or CM-κ-Car surface-functionalized 3D-printed PCL scaffolds did affect cell proliferation in a later stage of culture, i.e., after 14 days. Surface charge and wettability have been shown to affect early stage mineralization and bone cell–calcium phosphate interactions (Bodhak et al., 2009). Moreover, PCL scaffold roughness affects cell attachment and growth with minimal loss of mechanical strength (Gupta et al., 2019). Therefore, the increased proliferation as a result of SCMC or CM-κ-Car surface-functionalization as we found in the current study might be explained by enhanced surface hydrophilicity, roughness, and charge.

ALP activity and collagen production are important for bone formation (Jin et al., 2021). We found that ALP activity of pre-osteoblasts on 3D-printed PCL scaffolds without and with surface-functionalization by SCMC or CM-к-Car was similar after 7 days. After 14, 21, and 28 days of culture, the pre-osteoblasts showed higher levels of ALP activity on surface-functionalized scaffolds, indicating enhanced osteogenic differentiation on surface-functionalized 3D-printed PCL scaffolds. This was expected since both sulfate and carboxymethyl groups enhance ALP activity of pre-osteoblasts (Nakaoka et al., 2010). We also found that PCL/CM-κ-Car scaffolds more strongly stimulate ALP activity compared to PCL/SCMC scaffolds after 28 days of culture. Moreover, our data showed that collagen production by pre-osteoblasts on SCMC or CM-к-Car surface-functionalized 3D-printed PCL scaffolds was increased compared to unfunctionalized scaffolds after 21 and 28 days of culture. This was expected since sulfate groups enhance osteogenic differentiation of pre-osteoblasts (Miyazaki et al., 2008). We found that the oxygen concentration on the surface of the strand in the PCL/SCMC scaffolds was higher compared to PCL/CM-κ-Car scaffolds, resulting in enhanced hydrophilicity and cell proliferation. Since increased hydrophilicity enhances cell proliferation and collagen production (Ghilini et al., 2021), our results suggest that PCL/SCMC scaffolds more strongly stimulate cell proliferation and collagen production compared to PCL/CM-κ-Car scaffolds. However, since surface-functionalization by CM-κ-Car more strongly enhanced ALP activity, surface-functionalization by both SCMC and CM-κ-Car seems promising for stimulating bone formation.

An important factor for bone tissue engineering scaffolds is their ability to enhance matrix mineralization. We found that surface-functionalized 3D-printed PCL scaffolds by SCMC or CM-к-Car facilitated matrix mineralization after 21 days. This increased matrix mineralization can be explained by the fact that surface-functionalization resulted in a more negative surface charge of the scaffolds due to the presence of carboxyl and sulfate groups, which enabled efficient interaction with calcium ions (Liu et al., 2014). We also found that surface-functionalization by CM-κ-Car resulted in more mineralization compared to SCMC, which might be due to the presence of more sulfate groups in the chemical structure of CM-κ-Car compared to SCMC, as well as to increased surface roughness of PCL/CM-κ-Car scaffolds compared to PCL/SCMC scaffolds. Therefore, our findings suggest that surface chemical composition, which determines surface ionic charge and zeta potential, as well as surface roughness, may be key factors in the regulation of pre-osteoblast proliferation and osteogenic activity, more than surface hardness and hydrophilicity.

## 5 Conclusion

In the present study, 3D-printed PCL scaffolds were successfully surface-functionalized by SCMC or CM-к-Car. Surface-functionalized 3D-printed PCL scaffolds did possess higher surface hydrophilicity, negative charge, roughness, and hardness than unfunctionalized scaffolds, and improved MC3T3-E1 pre-osteoblast adhesion, proliferation, ALP activity, collagen production, and matrix mineralization. SCMC is superior to CM-к-Car in promoting pre-osteoblast proliferation, but CM-к-Car has an important advantage over SCMC in promoting osteogenic differentiation of pre-osteoblasts, suggesting surface-functionalization by CM-κ-Car may be more promising, especially in the short-term, for *in vivo* bone formation.

Our scaffolds have to be further studied regarding efficient vascularization for successful clinical implementation. Moreover, future research will have to elucidate whether these relatively small 3D-printed scaffolds can also be used in large critical sized bone defects without changing physicochemical characteristics and bioactivity. Finally, in view of clinical application of these surface-functionalized 3D-printed PCL scaffolds, their ability to promote cell behavior and bone formation should be investigated in bioreactors, *in-situ*, and *in vivo*.

## Data Availability

The data that support the findings of this study are openly available in the Figshare repository: https://figshare.com/s/acc742db2687855bed2d.
